# Brillouin Optomechanics in Coupled Silicon Microcavities

**DOI:** 10.1038/srep43423

**Published:** 2017-03-06

**Authors:** Y. A. V. Espinel, F. G. S. Santos, G. O. Luiz, T. P. Mayer Alegre, G. S. Wiederhecker

**Affiliations:** 1 Gleb Wataghin Physics Institute, University of Campinas, 13083-859 Campinas, SP, Brazil

## Abstract

The simultaneous control of optical and mechanical waves has enabled a range of fundamental and technological breakthroughs, from the demonstration of ultra-stable frequency reference devices, to the exploration of the quantum-classical boundaries in optomechanical laser-cooling experiments. More recently, such an optomechanical interaction has been observed in integrated nano-waveguides and microcavities in the Brillouin regime, where short-wavelength mechanical modes scatter light at several GHz. Here we engineer coupled optical microcavities to enable a low threshold excitation of mechanical travelling-wave modes through backward stimulated Brillouin scattering. Exploring the backward scattering we propose silicon microcavity designs based on laterally coupled single and double-layer cavities, the proposed structures enable optomechanical coupling with very high frequency modes (11 to 25 GHz) and large optomechanical coupling rates (*g*_0_/2*π*) from 50 kHz to 90 kHz.

Brillouin scattering occurs due to the interaction of optical and mechanical waves and it leads to the inelastic scattering of pump photons to Doppler red-shifted (Stokes) or blue-shifted (anti-Stokes) photons. In optical waveguides and microcavities this interaction occurs due to a combination of the photo-elastic effect^1^, induced by strain, and moving-boundary effect caused by the mechanical mode distortion of the optical boundaries^2^. These two scattering processes are strongly influenced by optical and mechanical properties of the confining structure and can be tailored for various applications. For instance, the generation of anti-Stokes photons, which is accompanied by destruction of phonon quanta, can be used to cool down mechanical modes in optical optomechanical cavities^3^,^4^; whereas the generation of stokes photons, which create phonons (heating), may foster the development of high-coherence lasers, ultra-stable radio frequency (RF) synthesizers^5^,^6^,^7^,^8^, and broadband tuning of RF filters^9^. Such confinement-enhanced optomechanical interaction has been observed as Stimulated Raman-like^10^ and Brillouin scattering in a range of photonic waveguides^11^,^12^,^13^,^14^,^15^,^16^,^17^,^18^ — where both energy and momentum conservation are directly fulfilled. In microcavities, however, the short roundtrip length and narrow linewidth further constrain the conservation laws, requiring both pump and scattered waves to be resonant with the optical cavity modes in order to ensure efficient Brillouin scattering. These constraints have limited the current cavity demonstrations of Brillouin scattering either to mm-scale cavities^5^,^19^,^20^,^21^, whose optical free-spectral range matches the mechanical resonant frequency; or heavily multimode micro-cavities, where the frequency difference of the distinct transverse optical modes accidentally matches the mechanical frequency, both at the cost of reduced optomechanical coupling.

Here we explore a compound microcavity system based on silicon microdisk cavities and demonstrate its potential to drastically enhances backward Brillouin scattering (BBS) for mechanical modes at tens of GHz. The compound microcavity scheme is illustrated in Fig. 1a and can ensure a doubly-resonant condition for the pump and stokes wave, yet preserving the small footprint necessary to achieve large optomechanical coupling. The individual cavities forming the compound system could be of various types, however, they must support mechanical modes whose frequencies can match the optical frequency splitting of the coupled modes. By engineering the mechanical modes, of single-disk (*sd*, Fig. 1b) and double-disk (*dd*, Fig. 1c) optical microcavities, to avoid cancellation between the photo-elastic (*pe*) and moving-boundary (*mb*) effect^22^, we demonstrate that both cavity designs could be exploited as components of the compound cavity scheme, thus offering a promising route towards the demonstration of low threshold backward stimulated Brillouin lasing in a compact silicon platform.

In backward Brillouin scattering the optical pump and the scattered Stokes waves propagate in opposite directions, resulting in a large wavevector mismatch that favors the interaction between light and short-wavelength propagating mechanical modes^1^. In disk microcavities, the optical and mechanical modes are azimuthally traveling waves with dependence exp(±*imϕ*) (here *m* is an integer and *ϕ* the azimuthal angle). Hence, a pump laser exciting an optical cavity mode with frequency and azimuthal number (*ω*_p_, *m*_p_) may be scattered into another optical mode (*ω*_s_, *m*_s_) through the interaction with a mechanical mode (Ω, *M*), provided that both energy and momentum (phase-matching) are conserved, i.e *ω*_s_(*m*_s_) = *ω*_p_(*m*_p_) ± Ω(*M*) and *m*_s_ = *m*_p_ ± *M*. While in forward Brillouin scattering the phase-matching condition favors mechanical modes close to their cut-off condition *M* = 0 (*m*_s_ = *m*_p_), in backward Brillouin scattering (BBS) the scattered light frequency shift is proportional to the optical wavevector mismatch and can easily reach tens of GHz in solids. In order to enhance BBS, such a large frequency shift would require the pump wave to be detuned from the optical resonance by tens or even hundreds of linewidth — *in a single-resonance cavity, such a large detuning would drop the benefits of the resonant cavity build-up for the pump wave*. In the proposed compound cavity system, illustrated in Fig. 1a, the interaction between the optical modes (through their evanescent fields) leads to a frequency splitting that can match the mechanical mode frequency. The outcome of this scheme is illustrated in Fig. 1d with the pump wave tuned to the higher frequency coupled mode at *ω*_p_, while the scattered wave is resonant with the lower frequency coupled mode at *ω*_s_, thus ensuring a high photonic density of states (PDOS) both at the pump and scattered frequencies (see Fig. 1e).

## Results

### Brillouin interaction

**Figure 1:**
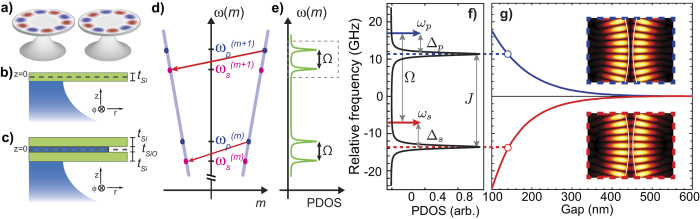
Backward Brillouin scattering in a compound microcavity system. (**a**) Schematic of the compound microcavity system based on microdisk cavities. The colorscale represents one optical coupled mode of the structure. Single-disk (**b**) and double-disk (**c**) cavity designs; *t*_Si_ = 250 nm, *t*_SiO_ = 100 nm, the Si radius corresponding to 5 micrometers. In the double-disk structure, the intermediate oxide layer has a radius of and 3.8 micrometers. (**d**) Optical dispersion diagram schematic, the optical resonances are represented by discrete (red and blue) points lying along the bulk dispersion curves (solid lines). Each pair of red and blue resonances are frequency split due to the lateral evanescent interaction in the compound system; the superscript *m* denotes the azimuthal order of each mode family. The arrows indicate possible resonant optical transitions from the pump (*ω*_p_) to the Stokes mode (*ω*_s_) due to BBS (**e**) Photonic density of states (PDOS) at the pump and scattered waves when the optical frequency splitting matches the mechanical mode frequency Ω. (**f**) Photonic density of states obtained for the coupled cavity modes with a quality factor of 10^5^ and splitting rate *J* = 25 GHz. The dashed blue and red lines indicate the optical mode resonant frequencies, 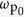, 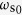, respectively. (**g**) Calculated optical frequency splitting. Blue and red curves indicate the anti-symmetric and symmetric coupled modes for a single-disk device. The insets show the calculated optical mode profiles for a 140 nm lateral air-gap between the cavities (circle’s labels).

The large azimuthal numbers involved in BBS imply that the phase-matched mechanical modes are localized near the cavity edge^23^,^24^,^25^, compared to low azimuthal number modes that are spread throughout the cavity; such an edge localization effectively increases the overlap between the optical and mechanical modes^26^. The mechanical mode induces strain and boundary deformation at the cavity edge, which efficiently Bragg scatter light and couples forward and backward propagating optical modes. The resulting energy exchange between the optical pump and Stokes wave can be modeled using coupled mode theory^27^,^28^, which leads to a set of coupled equations for the amplitudes of the pump, stokes, and mechanical waves (see Methods). We use the coupled mode theory to derive the threshold power necessary to achieve stimulated Brillouin lasing, which occurs when the Stokes photons gain, induced by the optical pump, suppresses the Stokes cavity mode loss. By assuming an undepleted pump the following expression can be derived for the input threshold power^27^ (see Supplementary Information),


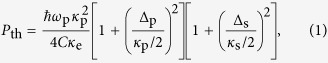


where 
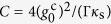
 is the so-called single-photon cooperativity; 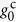 is the vacuum optomechanical coupling rate for the compound cavity, Γ is the mechanical loss rate 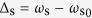 and 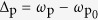 are the pump and stokes detunings relative to the cavity resonance doublets (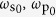), as illustrated in Fig. 1f; *κ*_s_ and *κ*_p_ are the corresponding total (intrinsic and extrinsic) loss rates, *κ*_e_ is the extrinsic pump loss rate due to coupling to the driving mode of the bus waveguide.

The lowest input threshold power is reached when both pump and Stokes waves are resonant with the mode doublet, (Δ_s_, Δ_p_) = 0. Since the Stokes photons are initially generated by spontaneous Brillouin scattering (due to thermally driven phonons), their frequencies are Doppler shifted *ω*_s_ = *ω*_p_ − Ω. When the pump and Stokes optical modes are split by a frequency difference *J*, their detuning is given by Δ_s_ = Δ_p_ + (*J* − Ω) (see Fig. 1f). Consequently, the threshold power scales as 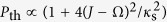, thus the minimum threshold occurs when the optical splitting precisely matches the mechanical frequency, i.e. *J* = Ω. Such a threshold power scaling reveals the importance of the doubly-resonant condition ensured by the compound cavity scheme. For instance, the minimum threshold power achievable using a standard single-resonance cavity occurs in the so-called resolved sideband limit (Ω_m_ ≫ *κ*), at an optimum pump detuning (Δ_p_ = Ω); this limit can be obtained from eq. 1: threshold assuming a large cavity separation (*J* → 0, then *κ*_s_ → *κ*_p_ and 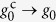). Hence a single-resonance cavity has a threshold power that is (Ω_m_/*κ*_p_)^2^ larger than the proposed compound cavity doubly-resonant approach. For a typical 5 µm radius microdisk (Ω_m_/*κ*_p_)^2^ ≈ 100, a roughly two-orders of magnitude higher threshold; where we assume an intrinsic quality factor of 2 × 10^5^ (*κ*_p,s_/2*π* ≈ 960 MHz) and Brillouin frequency Ω/2*π* = *V*_l_*M/r* ≈ 22 GHz — where *M/r* ≈ 4*π(n*_eff_/*λ*) and *n*_eff_ = 1.73 for the transverse-magnetic (TM) optical mode phase index (*λ* = 1.55 m), *V*_l_ is the Si bulk dilatational wave velocity.

Such high mechanical frequencies at tens of GHz can also be readily matched to the optical resonance splitting accessible by employing either single or double-disk silicon cavities. For example, we show in Fig. 1g the numerically calculated frequency splitting curves (solid lines) for a compound microcavity based on silicon single disks (see Methods), demonstrating splitting at tens of GHz for a lateral gap of 100 nm. This contrasts with larger and lower refractive index coupled microcavities whose frequency splitting lies in the sub-GHz range^29^ and could not enable resonant backward Brillouin scattering.

### Device design

**Figure 2:**
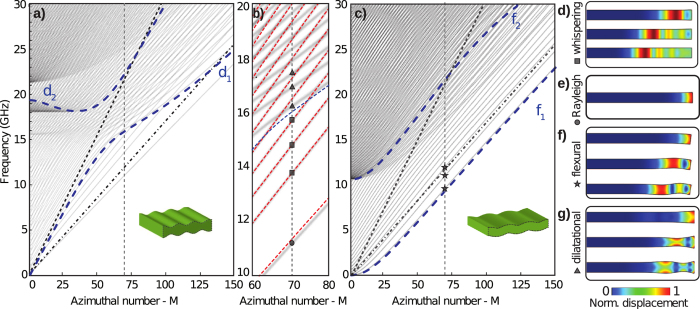
Mechanical dispersion in the *sd*-cavity. (**a**) Dispersion diagram for even modes (grey curves). The dashed and dash-dotted black solid lines represent the longitudinal (*V*_l_ = 9660 m/s) and transverse (*V*_t_ = 5340 m/s) bulk Si acoustic velocities. The dashed blue lines represent the dispersion of the first two dilatational modes (d_1_ and d_2_) of a 250 nm thick silicon slab (inset shows the d_1_ mode at *M* = 70). The vertical dashed line (*M* = 70) indicates the phase-matching azimuthal number for the TM optical mode in 1550 nm, *m*_p_ = *M*/2 = 35 for a disk radius of 5um. (**b**) Zoom for the dispersion of the even modes around of *M* = 70. The red dashed lines represent the dispersion of the whispering gallery modes for an infinite cylinder with the same radius. The blue dashed line is the dispersion of the slab d_1_ mode. The geometrical markers along to the vertical dashed identifies distinct modes groups in the *sd*-cavity. (**c**) Dispersion diagram for the odd modes (grey curves). The dashed blue lines represent the dispersion of the first two flexural modes (f_1_ and f_2_) of a 250 nm thick silicon slab, inset shows the slab f_1_ mode at *M* = 70 (The vertical dashed line). (**d**) First modes of the whispering gallery family (squares markers in **b**). (**e**) Rayleigh mode (circle marker in **b**). (**f**) First modes of the flexural family (star markers in **c**). (**g**) First modes of the dilatational family (triangle markers in **b**).

We demonstrate the feasibility of our compound cavity scheme by investigating two designs that can achieve high optomechanical coupling rates and mechanical frequencies at tens of GHz, the *sd* and *dd* cavities shown in Fig. 1b,c. Both designs could be readily fabricated^30^ and enhance the optomechanical interaction for distinct mechanical mode families. The frequency dispersion is the starting point to infer general characteristics of the phase-matched mechanical modes leading to backward Brillouin scattering. Many aspects of the mechanical modes dispersion of *sd* and *dd* cavities can be regarded as mixtures among whispering gallery mechanical modes of an infinite cylinder, and Lamb-wave modes of a free-standing silicon slab^23^,^31^. The mechanical mode dispersion of even and odd modes (with respect to *z* = 0 plane — see Fig. 1b) of a single disk are shown in Fig. 2. The dispersion curves were calculated using an axisymmetric finite element method (see Methods). In the *dd*-cavities, the mechanical modes are approximately symmetric/anti-symmetric combinations of the even and odd parity *sd*-cavity modes, therefore, the key characteristics of both designs may be inferred by inspecting only the *sd*-cavity mode structure.

The mechanical modes of *sd* cavities that may efficiently interact with optical modes can be divided in four groups: whispering gallery (*w*-modes), Rayleigh (*r*-modes), dilatational (*d*-modes), and flexural modes (*f*-modes). Their dispersion curves are shown in Fig. 2(a–c), and signaled by markers in Fig. 2(b,c); the corresponding displacement profiles are shown in Fig. 2(d–g). The whispering gallery group (*w*-modes) modes are remarkably similar to the modes of an infinite cylinder, as suggested by the excellent agreement between their dispersion curves (red-dashed lines), shown in the zoomed dispersion diagram of Fig. 2b. Despite the very small thickness/radius ratio of our disk (*t/r* = 0.05), the mechanical displacement components of *w*-modes are also similar to infinite cylinder modes, essentially in the radial-azimuthal (*rϕ*) directions. The peak displacement of increasing frequency *w*-modes shifts radially inward, which suggests a varying overlap with the optical mode.

The Rayleigh (*r*-modes), dilatational (*d*-modes), and flexural (*f*-modes) mode groups are signatures of the thin disk; their dominant radial-vertical (*rz*) displacement are noticeable in Fig. 2e–g. The *r*-mode is a singleton group and has the lowest frequency dispersion branch in Fig. 2a, which is characterized by a phase velocity lower than both the longitudinal (*V*_l_) and transverse (*V*_t_) bulk velocities^23^,^31^. Such a surface wave localization, noticeable in the displacement profile of Fig. 2e, compromises its overlap with the optical mode.

The dilatation (*d*-modes) originate from the tight vertical confinement of our thin disks. These characteristics are evidenced not only by their displacement profiles, shown in Fig. 2g, but also through their matching dispersion characteristics, highlighted in Fig. 2b (blue-dashed curve). The onset of the distinct disk mechanical mode families in Fig. 2a is also well matched by slab-mode cutoff frequency. Based on their displacement profile, the *d*-mode group is likely a strong candidate to large overlap with optical modes.

On the other hand, the *f*-group modes are cantilever-like modes and exhibit and odd symmetry relative to the *z* = 0 plane (see Fig. 1b), resulting in a negligible interaction with *sd*-cavity optical modes. In the *dd*-cavities, however, the symmetric combination of upper and lower-disk *f*-modes strongly modulate the air-gap between the disks, thus having potential to strongly interact with the *dd*-cavity optical modes. Indeed, these symmetric *f*-modes are similar to those explored in previous double-disk devices^32^,^33^, but due to their large azimuthal number they can readily vibrate in the 10 GHz frequency range.

### Optomechanical coupling

**Figure 3:**
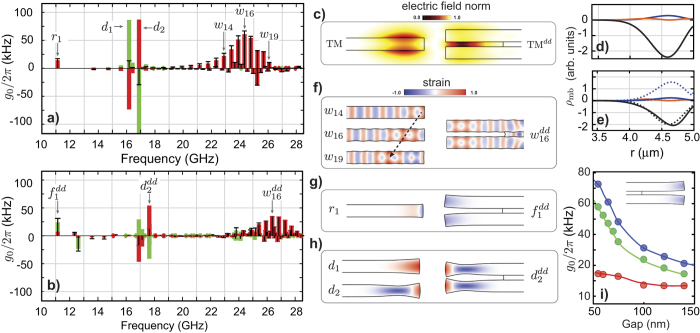
Optomechanical coupling in *sd* and *dd*-cavities. (**a**) and (**b**) Optomechanical coupling rate (black bars) between the optical mode and the mechanical even modes (respect to z = 0 plane in Fig. 1b,c) generated by the photoelastic *g*_pe_/2*π* (red) and moving boundary *g*_mb_/2*π* (green) contributions in the *sd* and *dd* cavities, respectively. (**c**) Electric field norm of the optical mode at 1550 nm for the *sd*-cavity (TM) and the *dd*-cavity (TM^*dd*^). (**d**) Contributions to the optical weighting function *ρ*_mb_ (eq. 2: gmb_terms) along the upper and lower *sd*-cavity boundaries. (**e**) Contributions to the optical weighting function along the outer (solid lines) and slot-interior (dashed-lines) *dd*-cavity boundaries. In (**d**) and (**e**) the line colors relate to each field component: red (*E*_*r*_), black (*E*_*ϕ*_) and blue (*D*_*z*_). (**f–h**) Dominant strain component (color scale) and deformation (amplified) for the mechanical modes labeled in part **a**) and **b**). The strain component *S*_*ϕϕ*_ is shown for the *w*-modes and *r*-modes (*r*_1_), *S*_*ϕz*_ is shown for *f*-modes (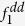) and *S*_*zz*_ is shown for the *d*-modes. (**i**) Optomechanical coupling rate for the 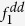 mode as a function of the gap between the upper and lower disk of the *dd*-cavity. The blue, green and red markers represent the total, *mb* and *pe* optomechanical coupling rates, respectively. The solid lines should only be considered as a guide.

The spatial overlap between optical modes and mechanical modes is necessary but not sufficient for a large optomechanical coupling. The optomechanical interaction in these high refractive index structures occurs due to a combination of the photo-elastic effect^1^ and deformation of the cavity boundaries^2^. The calculated optomechanical coupling rate must consider both effects, *g*_0_ = *g*_pe_ + *g*_mb_, where *g*_pe_ stands for the volumetric photo-elastic contribution (*pe*-effect), and *g*_mb_ for the cavity moving-boundaries contribution (*mb*-effect). In Fig. 3a,b we show the calculated photo-elastic (*g*_pe_, red), the moving-boundary (*g*_mb_, green) and total coupling rate *g*_0_ (bars) for the phase-matched mechanical modes in the single (Fig. 3a) and double-disk (Fig. 3b) structures. We focus on the mechanical modes that are phase-matched with fundamental TM (transverse magnetic) optical mode (Fig. 3c) since the TM-modes exhibits the largest coupling rate and potentially higher optical quality factors^34^.

For both *sd* and *dd*-structures, the whispering, Rayleigh, and dilatational mode groups can be identified in Fig. 3a,b. The relative contributions from the *pe* and *mb*-effects, however, varies significantly for each structure and mode group. To understand this in detail, we analyze the weighting function role played by the optical field in the *mb* and *pe*-effects (see Methods). For the *mb*-effect, the normal mechanical displacement (*u*_⊥_) is weighted by the optical field as (see Supplementary Information),


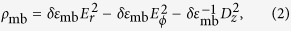


where *E*_*r*_, *E*_*ϕ*_ and *E*_*z*_ are the energy-normalized electric field components, *δε*_mb_ = *ε*_1_ − *ε*_2_ and 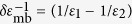 with 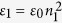 and 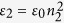 being the permittivities of silicon and air, respectively. The spatial dependence of the three terms in Eq. 2: gmb_terms are shown in Fig. 3d,e for both *sd* and *dd*-cavities. It is evident that the *mb*-effect is dominated by the azimuthal component (−*δε*_mb_|*E*_*ϕ*_|^2^) in both structures. The opposite sign of the azimuthal term relative to the radial contribution — due to the *π* phase difference between forward and backward azimuthal field components — substantially distinguishes the backward from the forward Brillouin optomechanical interaction. The weighting terms peaks around *r* = 4.6 µm also hint which mechanical modes should benefit from the *mb*-effect.

The photo-elastic (*pe*-effect) contribution is a bit more intricate due to the anisotropic nature of the strain-induced permittivity perturbation. Each component is calculated from the permittivity perturbation tensor, defined as 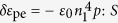, in which *p* is the photoelastic tensor of the isotropic silicon and *S* is the strain tensor induced by the mechanical waves (see Methods). For TM optical mode, however, the main permittivity perturbations are the vertical (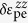) and azimuthal (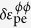) components since they are weighted by the dominant fields *E*_*z*_ and *E*_*ϕ*_. Despite the complexity of the *pe*-effect, the dominant photo-elastic coefficient in silicon is *p*_11_, hence an insight about which modes will lead to a strong *pe*-effect can be obtained analyzing only the azimuthal (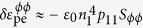) and vertical (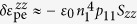) perturbations.

The *w*-mode group has the largest optomechanical coupling rate and give rise to several peaks in the high frequency range (20 ~ 28 GHz), for both *sd* (Fig. 3a) and *dd* cavities (Fig. 3b), which are unique to BBS due to the large mechanical azimuthal numbers imposed by the phase-matching condition. Due to their dominant displacement components (*u*_*r*_, *u*_*ϕ*_), the largest strain components is azimuthal, *S*_*ϕϕ*_ ≈ *Mu*_*ϕ*_/*r*. Such a large azimuthal strain leads to a *pe*-effect dominated optomechanical coupling, reaching the highest coupling rate for the single-disk, *g*_0_/2*π* ≈ 61 kHz at 24.34 GHz for the 16^th^ radial order *w*-mode (*w*_16_); the strain profile of selected *w*-modes are shown in Fig. 3f. In this mode group, 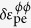 accounts for about 70% of the total coupling coefficient (see Supplementary Information, Table S2). The double-disk counterpart of this mode family (*w*^dd^) behaves similarly, yet with a reduced strength (*g*_0_/2*π* ≈ 35 kHz for the 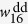) due to the larger optical and mechanical mode volumes. In either designs, the tiny in-plane displacement of this mode group always ensures a negligible contribution from the *mb*-effect.

Another interesting feature of this mode group is the peaked *g*_0_ distribution in Fig. 3(a,b), which can be understood by inspecting the overlap between the dominant azimuthal strain component *S*_*ϕϕ*_ (Fig. 3f) and the optical mode profile (Fig. 3c). Despite the strain oscillations along the radial direction, there is a net tensile strain (*S*_*ϕϕ*_ > 0) region indicated by the dashed arrow in Fig. 3f; as the mechanical frequency increases, the net strain region shifts radially inwards and sweeps the spatial matching between the strain and optical fields. The fine mechanical frequency spacing between adjacent *w*-modes and such a sweeping overlap lead to the observed peaked *g*_0_ distribution in Fig. 3(a,b). The physical origin of this net positive strain region relies on the hybrid longitudinal-transverse nature of the *w*-group, which can be precisely traced using the analytical solution of an infinite cylinder: the fast radial oscillation periods seen in Fig. 3f arise from the transverse-wave contribution to this mode, whereas the more slowly varying net positive strain regions are caused by longitudinal-wave contribution (see Supplementary Information, Fig. S4).

The Rayleigh mode (*r*_1_), which has the lowest resonant frequency (at 11.12 GHz, Fig. 3a), has a dominant radial displacement (*u*_*r*_) in the *sd*-cavity, which results in the edge-localized radial strain shown in Fig. 3g. On the other hand, its flexural odd counterpart in the *dd*-cavity, 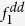, has also a large vertical (*u*_*z*_) component. Combined with the large azimuthal component, this leads to a dominant shear *S*_*ϕz*_ strain, which is shown in Fig. 3g. Due to its edge localization, away from the optical mode maximum, a minor role from the *mb*-effect is expected for the *r*_1_ mode in the *sd*-cavity. However, the boundary contribution for 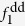 in the *dd*-cavity is outstanding, indeed this mode (11.15 GHz) has the second largest coupling rate among all the *dd*-cavity modes, reaching *g*_0_/2*π* = 31 kHz. Furthermore, the *dd*-cavity has a unique feature as its *mb*-effect strength can be tailored by adjusting the slot height between the two disks; in Fig. 3i we show that the total coupling rate (*g*_0_) of the 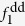 mode can be improved by 300% by reducing the slot height from 150 nm to 50 nm.

Finally, a very high optomechanical coupling rate could be expected for the *d*-mode group (16 GHz  < Ω/2*π* < 18 GHz). These modes are striking examples that intuition based on mode shapes may fail when predicting the BBS interaction strength in microstructures. The *d*-modes display not only a large vertical strain but also a large deformation of the cavity boundaries, as shown in Fig. 3h. Indeed, these two effects are very strong individually but their opposite sign lead to a cancellation effect, a clear competition between the *mb (g*_mb_) and *pe*-effects (*g*_mb_)^22^. For the double-disk structure, whose optical weighting function is shown in Fig. 3e, the slot effect enhancement does not readily improve the optomechanical coupling with the dilatational modes, this is due to a balanced contribution from the azimuthal (dashed black line) and vertical field (dashed blue line) components along the slot boundaries, which oppositely contribute to the *mb*-effect and almost cancel it.

## Discussion

The calculated BBS coupling rates for both *sd* and *dd*-cavities improve over a few orders of magnitude compared to coupling rates reported for larger silica microspheres (*g*_0_/2*π* ≈ 20 Hz)^35^ and crystalline resonators (*g*_0_/2*π* ≈ 61 Hz)^20^. Such high coupling rates ensure that the proposed designs will have a low input power threshold to achieve stimulated Brillouin lasing, despite the lower quality factors often achievable with silicon cavities. For instance, we assume an optical mode resonant around the wavelength of 1550 nm, with an intrinsic optical quality factor of 2 × 10^5^ (*κ*_p_/2*π* = *κ*_s_/2*π* ≈ 1.2 GHz), and mechanical quality factor of 10^3^, which are conservative optical and mechanical mode parameters. If these cavities designs are employed in the proposed compound cavity scheme, and the mechanical frequency matches the optical frequency splitting, i.e., (Δ_s_ = Δ_p_ = 0) in Eq. 1: threshold. The predicted threshold power for SBS lasing is only *P*_th_ ≈ 8 mW for the single-disk *w*_16_ mode; *P*_th_ ≈ 31 mW for the double-disk 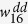 mode.

For the cantilever-like flexural mode of the *dd*-cavity (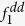), the threshold power is *P*_th_ ≈ 17 mW (assuming the same mechanical quality factor). This threshold power for the 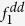-mode can be reduced even further for smaller double-disk separation. For instance, if *t*_SiO_ = 50 nm, it is possible to achieve *g*_0_/2*π* ≈ 75 kHz (Fig. 3i), leading to a threshold power of only *P*_th_ ≈ 3 mW (considering the same optical and mechanical losses).

Experimentally, in order to ensure the simultaneous resonant condition, a set of coupled optical cavities with varying coupling gaps could be fabricated. The importance of the compound cavity scheme also becomes clear if we compare the results above with a single cavity (single or double-disk) input threshold power, which is predicted by eq:threshold assuming a resonant stokes signal Δ_s_ = 0 and the optimal pump-detuning (Δ_p_ = Ω_m_). Using the same optical and mechanical linewidth above, and a single-photon optomechanical coupling rate (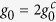, see Methods), the threshold for Brillouin lasing would be as high as *P*_th_ ≈ 3.2 W for the *w*_16_, and *P*_th_ ≈ 12.3 W for the 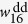 mode. Such a high input powers are rather impractical due to strong detrimental effects they induce, such as strong nonlinear light absorption or even thermal damage of the bus waveguides.

## Conclusions

Our results provide a clear guideline towards the observation of backward Brillouin scattering lasing in integrated CMOS-compatible silicon cavities. Both the single-disk and double-disk designs were shown to enable large optomechanical coupling rates, a few orders of magnitude larger than previously reported resonators, allowing for mW-level input power lasing thresholds. The single-disk device, despite its simplicity, was shown to enable mechanical modes at very high frequencies (25 GHz) with large optomechanical coupling rates. The double-disk device, although exhibiting a lower optomechanical coupling for high frequency modes, was shown to drastically enhance the interaction with flexural modes around 11 GHz. Although we concentrated our discussion on silicon-based devices, our results could be adapted to similar structures fabricated from other high index materials, such as III-V, Si_3_N_4_ and SiO_2_.

## Methods

### Frequency splitting

The frequency splitting simulation was performed using a two-dimensional approximation to the actual *sd*-cavity. In this approximation, the modes of the coupled infinite cylinders are calculated while constraining the out-of-plane wave number,


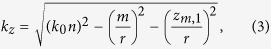


where *m/r* and *z*_*m*,1_/*r* are the azimuthal and radial components of the wave vector with norm *k*_0_*n, k*_0_ is the free-space wave number for *λ* = 1.55 µm, *n* = 3.5 is the silicon refractive index, *r* = 5 µm, *m* = 35 is the optical azimuthal number for the TM-mode of the *sd*-cavity and *z*_*m*,1_ is the first zero of the Bessel function *J*_*m*_(*z*). This is equivalent to the Marcatilli effective index method and we verified that it leads to an electric field envelope that agrees well with the numerical mode obtained from the axisymmetric calculation.

#### Mechanical mode dispersion

We obtain the dispersion relation Ω(*M*) by solving the eigenfrequency problem derived from the full-vectorial elastic wave equation through the finite-element method (see Supplementary Information), due to the highly multimode character of the mechanical dispersion, we show in Fig. 2a–c a grey color shading proportional to the mechanical density of modes instead of the calculated pairs (Ω_*M*_, *M*). The mechanical density of modes is calculated as 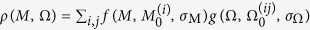, where *f* and *g* are Gaussian weighting functions with a normalized product, full-width-half-maximum (FWHM) wavenumber *σ*_M_ = 0.1, FWHM-frequency *σ*_Ω_/2*π* = 117.5 MHz and given one azimuthal acoustic number 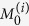 are calculated each of the frequencies 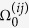 from the elastic wave equation.

#### Coupled mode equations

The resulting energy exchange between the optical pump and Stokes wave can be modeled using coupled mode theory^27^,^28^ (see Supplementary Information), which leads to a set of coupled equations for the amplitudes of the pump wave (*a*_p_), stokes wave (*a*_s_) and mechanical wave (*b*),


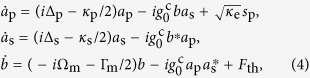


where *a*_i_’s are normalized such that |*a*_i_|^2^ is the intra-cavity photon number and *b* is normalized such that |*b*|^2^ is the phonon number. 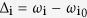 is the frequency detuning between the pump (i = p) or stokes wave (i = s) relative to the optical cavity mode frequencies 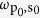 . *κ*_p,s_ represents the optical loss rate for each mode, (Ω_m_,Γ_m_) are the mechanical mode frequency and damping rate, respectively; the optomechanical coupling rate is 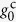 and represents the photon coupling rate between the stokes and the pump wave induced by the zero-point fluctuation of the mechanical mode, which will be calculated shortly using electromagnetic perturbation theory^2^,^36^. Note that 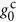 is related to the more usual single-cavity coupling rate as 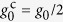. The factor 1/2 arises because the coupled optical mode is distributed within two optical cavities whereas the mechanical mode is localized within a single cavity due to the air gap between the cavities. *κ*_e_ is extrinsic coupling rate to the feeding waveguide carrying a photon-flux |*s*_p_|^2^. *F*_th_ is a white-noise random thermal force responsible for driving the mechanical motion.

#### *g*_0_ calculation

The *mb*-effect contribution is given by ref. 2 (see Supplementary Information),


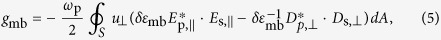


where the permittivity differences are given by *δε*_mb_ = *ε*_1_ − *ε*_2_ and 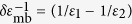, in which 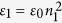 and 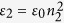 are the permittivities of the silicon and air, respectively. 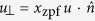 is the surface-normal component of the displacement vector *u* (normalized to unit); 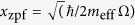 is zero-point fluctuation of the mechanical mode with effective mass *m*_eff_; the fields 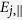 and *D*_*j*,⊥_ are boundary-tangential electric field and boundary-normal electric displacement field to the cavity surface *S* of the pump (*j* = p) or scattered (*j* = s) optical mode (energy-normalized). The *pe*-effect contribution is given by ref. 36 (see Supplementary Information),


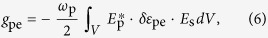


where 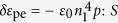 is the photo-elastic perturbation in the permittivity inside the cavity volume *V, p* is the photoelastic tensor of silicon, and *S* = *x*_zpf_∇_*s*_*u* is the strain tensor induced by the mechanical waves. The optical and elastic mode profiles are numerically calculated using the finite-element method (see Supplementary Information).

#### Simulation parameters

Si refractive index *n*_Si_ = 3.5, silica refractive index 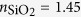, air refractive index *n*_air_ = 1.0, wavelength of interest *λ* = 1550 nm, Si Young’s modulus *E*_Si_ = 170 GPa, silica Young’s modulus 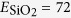 GPa, Si Poisson’s ratio *ν*_Si_ = 0.28, silica Poisson’s ratio 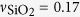, Si density mass *ρ*_Si_ = 2329 kg/m^3^, silica density mass 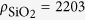 kg/m^3^ and Si photo-elastic coefficients^37^ *p*_11_ = −0.09, *p*_12_ = 0.017 and *p*_44_ = −0.0535. Here we neglect silicon anisotropy and assume the values of *E*_Si_ and *ν*_Si_ along to principal crystal axes^38^.

## Additional Information

**How to cite this article:** Espinel, Y. A. V. *et al*. Brillouin Optomechanics in Coupled Silicon Microcavities. *Sci. Rep.* **7**, 43423; doi: 10.1038/srep43423 (2017).

**Publisher's note:** Springer Nature remains neutral with regard to jurisdictional claims in published maps and institutional affiliations.

## Supplementary Material

Supplementary Information
