# Supplementary material for: Brillouin Optomechanics in Coupled Silicon Microcavities
Source: Sci Rep. 2017 Mar 6;7:43423. doi: 10.1038/srep43423 (PMC5338249; doi:10.1038/srep43423)
Supplement: Supplementary Information [file srep43423-s1.pdf]

Supplementary Information: Brillouin Optomechanics in Coupled Silicon Microcavities

Y. A. V. Espinel,[1] F. G. S. Santos,[1] G. O. Luiz,[1] T. P. M. Alegre,[1] and G. S. Wiederhecker[1,*]

[1] *Gleb Wataghin Physics Institute, University of Campinas, 13083-970 Campinas, SP, Brazil*

(Dated: December 20, 2016)

* gustavo@ifi.unicamp.br

S1. COUPLED MODE EQUATIONS

We derive the coupled mode equations for the optical and mechanical modes following an approach similar to [1]. The electric field is obtained from Maxwell's wave equation in the presence of a time-dependent polarization term,

$$\nabla \times \nabla \times \boldsymbol{\mathcal{E}} = -\mu_0 \epsilon \partial_t^2 \boldsymbol{\mathcal{E}} - \mu_0 \partial_t^2 (\delta \boldsymbol{P}), \tag{S1}$$

where $\boldsymbol{\mathcal{E}}$ is total electric field vector, μ_0 is the vacuum permeability, ϵ is the isotropic unperturbed spatial permittivity. The additional polarization, $\delta \boldsymbol{P}$, arises from the mechanical mode perturbation to the optical field. The mechanical modes are described by the equation of motion,

$$\nabla \cdot (\boldsymbol{c} : \boldsymbol{\mathcal{S}}) - \rho\, \partial_t^2 \boldsymbol{\mathcal{U}} = -\boldsymbol{\mathcal{F}}, \tag{S2}$$

where $\boldsymbol{\mathcal{U}}$ is the mechanical displacement, $\boldsymbol{c}$ is the stiffness tensor, $\boldsymbol{\mathcal{S}} = \nabla_{\mathrm{s}} \boldsymbol{\mathcal{U}}$ is the strain tensor, ρ is the material density, and $\boldsymbol{\mathcal{F}}$ is the force density vector with contributions from the electric part of the Maxwell stress tensor and electrostriction tensor [2].

Optical equations

To obtain the coupled mode equations for the optical fields we expand $\boldsymbol{\mathcal{E}}$ in terms of slowly-varying amplitudes for the pump (p) and stokes (s) fields, we consider the modal expansion,

$$\boldsymbol{\mathcal{E}}(\boldsymbol{r}, t) = \sum_{j=\mathrm{p,s}} a_j(t) e^{-i\,\omega_j\,t} \boldsymbol{E}_j(\boldsymbol{r}) + c.c. \tag{S3}$$

The optical mode spatial distribution $\boldsymbol{E}_j(\boldsymbol{r})$ is normalized such that $\sum_j |a_j|^2$ represents the total optical energy. Each modal fields satisfy the Helmholtz equation,

$$\nabla \times \nabla \times \boldsymbol{E}_j = \omega_{0,j}^2\, \mu_0\, \epsilon \boldsymbol{E}_j, \tag{S4}$$

where $\omega_{0,j}$ is the resonant frequency of each optical mode. These modes are orthonormalized,

$$\int \boldsymbol{E}_m^* \cdot \epsilon \boldsymbol{E}_n \, dV = \delta_{m,n}. \tag{S5}$$

Substituting eq. (S3) in eq. (S1), exploring the slowly-varying envelope approximation (SVEA) $(d/dt \ll \omega_j)$ and the small detuning approximation, $\omega_j^2 - \omega_{0,j}^2 \approx 2\omega_j \Delta_j$ (with $\Delta_j = \omega_j - \omega_{0,j}$) we arrive at the following coupled equations for the field amplitudes a_j,

$$\sum_j \left[2\omega_j \left(i\dot{a}_j + \Delta_j a_j\right)\right] e^{-i\omega_j t} \epsilon \boldsymbol{E}_j + c.c. = \partial_t^2 (\delta \boldsymbol{P}). \tag{S6}$$

We can decouple eq. (S6) by multiplying it by $\boldsymbol{E}_l^*$, integrating over the whole space, and using eq. (S5),

$$\left(i\dot{a}_l + \Delta_l a_l\right) e^{-i\omega_l t} + c.c. = \frac{\int [\boldsymbol{E}_l^* \cdot \partial_t^2 (\delta \boldsymbol{P})] dV}{2\omega_l}. \tag{S7}$$

The spatial and time-dependence of the polarizability is given by,

$$\delta \boldsymbol{P}(\boldsymbol{r}, t) = \delta \boldsymbol{\varepsilon}(\boldsymbol{r}, t) \cdot \boldsymbol{E}(\boldsymbol{r}, t), \tag{S8}$$

where the time-dependence of the permittivity perturbation $\delta \boldsymbol{\varepsilon}(\boldsymbol{r}, t)$ will be given by the mechanical mode,

$$\boldsymbol{\mathcal{U}}(\boldsymbol{r}, t) = b(t) e^{-i\Omega t} \boldsymbol{u}(\boldsymbol{r}) + c.c., \tag{S9}$$

where we choose to normalize the mechanical spatial distribution such that $\max(|\boldsymbol{u}(\boldsymbol{r})|) = 1$, therefore $b(t)$ has units of length.

The mechanical mode will perturb the optical mode both through the photo-elastic (pe) effect and through of the moving boundary (mb) effect. Either contributions will be proportional to the displacement amplitude $b(t)$. Therefore the permittivity perturbation time-dependence can be factored out as $\delta\boldsymbol{\varepsilon}(\boldsymbol{r},t) = (b(t)/b_0)\exp(-i\Omega t)\delta\boldsymbol{\epsilon}(\boldsymbol{r}) + c.c.$, where $\delta\boldsymbol{\epsilon}(\boldsymbol{r})$ is the spatial permittivity perturbation and b_0 is a free-parameter of amplitude normalization with units of length. Substituting this expression together with eq. (S3) into eq. (S8) we obtain,

$$\delta\boldsymbol{P}(\boldsymbol{r},t) = \left(\frac{1}{b_0}\right)\left(b(t)e^{-i\Omega t}\delta\boldsymbol{\epsilon}(\boldsymbol{r}) + c.c\right)\cdot\left(\sum_m a_m(t)e^{-i\omega_m t}\boldsymbol{E}_m(\boldsymbol{r}) + c.c.\right). \tag{S10}$$

There will be four distinct terms for each term m in the summation of eq. (S10),

$$b(t)a_m(t)\delta\boldsymbol{\epsilon}(\boldsymbol{r})\cdot\boldsymbol{E}_m(\boldsymbol{r})e^{i(-\omega_m-\Omega)t} + b^*(t)a_m^*(t)\delta\boldsymbol{\epsilon}^*(\boldsymbol{r})\cdot\boldsymbol{E}_m^*(\boldsymbol{r})e^{i(\omega_m+\Omega)t}$$
$$+b(t)a_m^*(t)\delta\boldsymbol{\epsilon}(\boldsymbol{r})\cdot\boldsymbol{E}_m^*(\boldsymbol{r})e^{i(\omega_m-\Omega)t} + b^*(t)a_m(t)\delta\boldsymbol{\epsilon}^*(\boldsymbol{r})\cdot\boldsymbol{E}_m(\boldsymbol{r})e^{i(-\omega_m+\Omega)t},$$

in a rotating-wave approximation (RWA) these distinct terms will be relevant drives to the eq. (S7) provided they satisfy the energy conservation. The energy-conserving terms will depend whether we are treating the pump (a_p) or Stokes (a_s) amplitudes. For example, for the Stokes wave $\omega_\mathrm{s} = \omega_\mathrm{p} - \Omega$ only the last term is relevant. The time-derivative of the polarization in eq. (S7) will have terms involving the first and second derivatives of the slowly varying amplitudes $a_m(t), b(t)$ and terms of the order of ω_m^2. Employing the SVEA and choosing the relevant driving terms from eq. (S10), we can finally write the amplitude equations for *positive frequency* amplitudes of the optical fields; dropping out the fast oscillating terms in both sides of eq. (S7) lead to,

$$\dot{a}_\mathrm{p} = i\,\Delta_\mathrm{p}\,a_\mathrm{p} - \frac{i\,g_0\,b\,a_\mathrm{s}}{b_0}, \tag{S11}$$

$$\dot{a}_\mathrm{s} = i\,\Delta_\mathrm{s}\,a_\mathrm{s} - \frac{i\,g_0^*\,b^*\,a_\mathrm{p}}{b_0}, \tag{S12}$$

where,

$$g_0 = -\frac{\omega_\mathrm{p}}{2}\int_V \boldsymbol{E}_\mathrm{p}^*\cdot\delta\boldsymbol{\epsilon}\cdot\boldsymbol{E}_\mathrm{s}\,dV \tag{S13}$$

represents the optomechanical coupling rate.

Mechanical equation

To find the equation of motion for the mechanical modes we proceed in a similar fashion. Each mechanical mode satisfies the modal equation,

$$\nabla\cdot(\boldsymbol{c}{:}\boldsymbol{S}) = -\rho\,\Omega_0^2\,\boldsymbol{u}, \tag{S14}$$

where $\boldsymbol{S} = \nabla_\mathrm{s}\,\boldsymbol{u}$ is the spatial distribution of the strain tensor per unit length and Ω_0 is the mechanical mode resonant frequency. Substituting the mechanical mode expansion eq. (S9) in eq. (S2) and, in the resulting equation, substituting eq. (S14) and exploring the small-detuning approximation $\Omega^2 - \Omega_0^2 \approx 2\,\Omega\,\Delta_\mathrm{m}$ (with $\Delta_\mathrm{m} = \Omega - \Omega_0$), we arrive at,

$$2\,\Omega(i\dot{b} + \Delta_\mathrm{m}b)e^{-i\,\Omega\,t} + c.c. = \frac{\langle\boldsymbol{u}|\boldsymbol{\mathcal{F}}\rangle}{m_\mathrm{eff}}, \tag{S15}$$

where $\langle\boldsymbol{u}|\boldsymbol{\mathcal{F}}\rangle = \int \boldsymbol{u}^*\cdot\boldsymbol{\mathcal{F}}\,dV$, $m_\mathrm{eff} = \int\rho|\boldsymbol{u}|^2dV$ is the effective motional mass, $\boldsymbol{\mathcal{F}} = \boldsymbol{\mathcal{F}}_\mathrm{MT} + \boldsymbol{\mathcal{F}}_\mathrm{ES}$, in which $\boldsymbol{\mathcal{F}}_\mathrm{MT} = \nabla\cdot\boldsymbol{\mathcal{T}}$ is the force density from the Maxwell stress tensor and $\boldsymbol{\mathcal{F}}_\mathrm{ES} = -\nabla\cdot\boldsymbol{\varsigma}$ is the force density from the electrostriction tensor,

$$\mathcal{T}_{ij} = \epsilon\left(\mathcal{E}_i\,\mathcal{E}_j - \frac{1}{2}\delta_{ij}|\boldsymbol{\mathcal{E}}|^2\right), \tag{S16}$$

$$\varsigma_{ij} = \gamma_{ijkl}\,\mathcal{E}_k\,\mathcal{E}_l, \tag{S17}$$

are the time-dependent electric Maxwell and electrostriction stress tensors, $\gamma_{ijkl} = -(1/2)\epsilon_0 n^4 p_{ijkl}$ with n being the optical refractive index and p_{ijkl} the photoelastic tensor. The minus sign used in the definition of the electrostrictive force follows the conservative force convention [2, 3]. Using our field expansion eq. (S3), the general form of the field products in eq. (S16) and eq. (S17) are in the form,

$$\mathcal{E}_i\mathcal{E}_j = \left(\sum_l a_l(t)e^{-i\omega_l t}E_l^{(i)} + c.c.\right)\left(\sum_m a_m(t)e^{-i\omega_m t}E_m^{(j)} + c.c.\right), \tag{S18}$$

where the parenthesis superscript (i, j) indicate the spatial component of the modal field. According to RWA, among all terms in eq. (S18) the only relevant ones are those oscillating at the mechanical frequency, i.e., terms with frequencies $\omega_\mathrm{p} - \omega_\mathrm{s}$. Therefore, substituting eq. (S18) in eq. (S16) and eq. (S17), considering the relevant terms,

$$\mathcal{T}_{ij} = a_\mathrm{p}\, a_\mathrm{s}^*\, T_{ij}\, e^{-i(\omega_\mathrm{p}-\omega_\mathrm{s})t} + c.c., \tag{S19}$$

$$\varsigma_{ij} = a_\mathrm{p}\, a_\mathrm{s}^*\, \sigma_{ij}\, e^{-i(\omega_\mathrm{p}-\omega_\mathrm{s})t} + c.c., \tag{S20}$$

where

$$T_{ij} = \epsilon[E_\mathrm{p}^{(i)}E_\mathrm{s}^{(j)*} + E_\mathrm{p}^{(j)}E_\mathrm{s}^{(i)*} - \delta_{ij}\boldsymbol{E}_\mathrm{p}\cdot\boldsymbol{E}_\mathrm{s}^*], \tag{S21}$$

$$\sigma_{ij} = \gamma_{ijkl}[E_\mathrm{p}^{(k)}E_\mathrm{s}^{(l)*} + E_\mathrm{p}^{(l)}E_\mathrm{s}^{(k)*}], \tag{S22}$$

are the spatial distributions of the electric Maxwell and electrostriction stress tensors, respectively. Therefore, substituting eq. (S19) and eq. (S20) in the driving term,

$$\langle \boldsymbol{u}|\boldsymbol{\mathcal{F}}\rangle = \int \boldsymbol{u}^*\cdot\nabla\cdot\boldsymbol{\mathcal{T}}\, dV - \int \boldsymbol{u}^*\cdot\nabla\cdot\boldsymbol{\varsigma}\, dV, \tag{S23}$$

and substituting the resulting equation in eq. (S15) and then time-averaging we obtain the dynamical equation for the mechanical mode amplitude,

$$\dot{b} = i\,\Delta_\mathrm{m}\, b + \frac{i\, a_\mathrm{p}\, a_\mathrm{s}^*}{2\,\Omega}\frac{\langle \boldsymbol{u}|\boldsymbol{f}\rangle}{m_\mathrm{eff}}, \tag{S24}$$

where

$$\langle \boldsymbol{u}|\boldsymbol{f}\rangle = \int \boldsymbol{u}^*\cdot\boldsymbol{f}\, dV = \int \boldsymbol{u}^*\cdot(\nabla\cdot\mathbf{T} - \nabla\cdot\boldsymbol{\sigma})\, dV. \tag{S25}$$

The relation between the coupling coefficients and optical forces

It is known that the electric Maxwell stress tensor lead only to a boundary force in a transparent material, whereas the electrostriction tensor leads to a volume force [4, 5]. These two contributions can be obtained by integrating by parts eq. (S25) and disregarding the electrostriction surface pressure term [3, 5],

$$\langle \boldsymbol{u}|\boldsymbol{f}\rangle = \oint_S \boldsymbol{u}^*\cdot\boldsymbol{f}_\mathrm{rp}\, dA + \int_V \boldsymbol{\sigma}:\boldsymbol{S}^*\, dV, \tag{S26}$$

where the double inner product is defined as $\boldsymbol{\sigma}:\boldsymbol{S}^* = \sigma_{ij}S_{ij}^*$, and,

$$\boldsymbol{f}_\mathrm{rp} = (\mathbf{T}_2 - \mathbf{T}_1)\cdot\hat{n}, \tag{S27}$$

represent the spatial distribution of the radiation pressure making on the surface S of the cavity with volume V. $\mathbf{T}_1$ and $\mathbf{T}_2$ are the Maxwell stress tensors calculated inside and outside of the cavity, respectively, and $\hat{n}$ is the unitary normal vector to S that points from inside to outside of the cavity.

A more convenient form of the radiation pressure is obtained when eq. (S21) is substituting in eq. (S27) considering two different materials and continuous fields on S,

$$\boldsymbol{f}_{\mathrm{rp}} = [\delta\epsilon_{\mathrm{mb}}(\boldsymbol{E}^*_{\mathrm{s},\parallel}\cdot\boldsymbol{E}_{\mathrm{p},\parallel}) - \delta\epsilon^{-1}_{\mathrm{mb}}(\boldsymbol{D}^*_{\mathrm{s},\perp}\cdot\boldsymbol{D}_{\mathrm{p},\perp})]\hat{n}, \tag{S28}$$

in which $\boldsymbol{E}_{p,\parallel}$ and $\boldsymbol{E}_{s,\parallel}$ are the parallel electric fields from pump and the Stokes waves, respectively, $\boldsymbol{D}_{p,\perp}$ and $\boldsymbol{D}_{s,\perp}$ are the perpendicular electric displacements from pump and Stokes waves, respectively, $\delta\epsilon_{\mathrm{mb}} = \epsilon_0(n_1^2 - n_2^2)$ and $\delta\epsilon^{-1}_{\mathrm{mb}} = \epsilon_0^{-1}(n_1^{-2} - n_2^{-2})$. As parallel electric fields and perpendicular electric displacements are normalized then the radiation pressure is also normalized such that it has units of L^{-3}, as can be evaluated from eq. (S28).

Like radiation pressure, second term in the right-hand of eq. (S26) can be also written in a more convenient form,

$$\int_V \boldsymbol{\sigma}:\boldsymbol{S}^*\,dV = \int_V (\boldsymbol{E}^*_{\mathrm{p}}\cdot\delta\boldsymbol{\epsilon}_{\mathrm{pe}}\cdot\boldsymbol{E}_{\mathrm{s}})^*\,dV, \tag{S29}$$

where $\delta\boldsymbol{\epsilon}_{\mathrm{pe}} = -\epsilon_0\,n^4\,\boldsymbol{p}:\boldsymbol{S}$ is the anisotropic perturbation in the permittivity per unit length from the photoelastic effect. Now substituting eqs. (S28) and (S29) in eq. (S26) results,

$$\langle\boldsymbol{u}|\boldsymbol{f}\rangle = \oint_S (\boldsymbol{u}^*\cdot\hat{n})[\delta\epsilon_{\mathrm{mb}}(\boldsymbol{E}^*_{\mathrm{s},\parallel}\cdot\boldsymbol{E}_{\mathrm{p},\parallel}) - \delta\epsilon^{-1}_{\mathrm{mb}}(\boldsymbol{D}^*_{\mathrm{s},\perp}\cdot\boldsymbol{D}_{\mathrm{p},\perp})]dA + \int_V (\boldsymbol{E}^*_{\mathrm{p}}\cdot\delta\boldsymbol{\epsilon}_{\mathrm{pe}}\cdot\boldsymbol{E}_{\mathrm{s}})^* dV, \tag{S30}$$

where both integrals has units of L^{-1}.

The coupled optomechanical equations

Finally, considering the transformations: $a_{\mathrm{p}} \to \sqrt{\hbar\omega_{\mathrm{p}}}\,a_{\mathrm{p}}$, $a_{\mathrm{s}} \to \sqrt{\hbar\omega_{\mathrm{s}}}\,a_{\mathrm{s}}$, $b \to b_0\,b$ in eqs. (S11), (S12) and (S24), assuming $\omega_{\mathrm{s}} \approx \omega_{\mathrm{p}}$ and imposing the Manley-Rowe conditions, we obtain the coupled mode equations in terms of the normalized amplitudes (a_{p}, a_{s}, b),

$$\dot{a}_{\mathrm{p}} = i\,\Delta_{\mathrm{p}}\,a_{\mathrm{p}} - i\,g_0\,b\,a_{\mathrm{s}}, \tag{S31}$$

$$\dot{a}_{\mathrm{s}} = i\,\Delta_{\mathrm{s}}\,a_{\mathrm{s}} - i\,g_0^*\,b^*\,a_{\mathrm{p}}, \tag{S32}$$

$$\dot{b} = i\,\Delta_{\mathrm{m}}\,b - i\,g_0^*\,a_{\mathrm{p}}\,a_{\mathrm{s}}^*, \tag{S33}$$

where,

$$g_0 = -\frac{\omega_{\mathrm{p}}}{2}\,\langle\boldsymbol{u}|\boldsymbol{f}\rangle^*\,x_{\mathrm{zpf}} = g_{\mathrm{om}}\,x_{\mathrm{zpf}}, \tag{S34}$$

if we consider $b_0 = 2\,x_{\mathrm{zpf}}$ [6]. According to eqs. (S30) and (S34), g_{om} (and g_0) can be decomposed as a sum of two contributions,

$$g^{\mathrm{pe}}_{\mathrm{om}} = -\frac{\omega_{\mathrm{p}}}{2}\int_V \boldsymbol{E}^*_{\mathrm{p}}\cdot\delta\boldsymbol{\epsilon}_{\mathrm{pe}}\cdot\boldsymbol{E}_{\mathrm{s}}\,dV, \tag{S35}$$

$$g^{\mathrm{mb}}_{\mathrm{om}} = -\frac{\omega_{\mathrm{p}}}{2}\oint_S [\boldsymbol{u}\cdot\hat{n}][\delta\epsilon_{\mathrm{mb}}[\boldsymbol{E}^*_{\mathrm{p},\parallel}\cdot\boldsymbol{E}_{\mathrm{s},\parallel}] - \delta\epsilon^{-1}_{\mathrm{mb}}[\boldsymbol{D}^*_{\mathrm{p},\perp}\cdot\boldsymbol{D}_{\mathrm{s},\perp}]]\,dA, \tag{S36}$$

representing the optomechanical coupling contributions from the strain the photo-elastic (pe) and moving-boundary (mb) effects.

S2. FINITE ELEMENT METHOD

The eigenvalues and eigenvectors for the optical and mechanical fields are solved by using finite elements method (FEM) applied to the Helmholtz equation (eq. (S4)) and the equation of motion (eq. (S14)), respectively, both

implemented in a commercial software (COMSOL 4.4). The optical modes are simulated by using the Electromagnetic Frequency Domain Interface (emw) with a modified weak form to ensure convenient field solutions whereas the mechanical modes are simulated by using the Weak Form PDE Interface (w). As the symmetry of the cavities suggest, we use cylindrical coordinates (2D-axisymmetric component in COMSOL) to simulate the structures.

We also assume a negligible effect of the pedestal on the optical and mechanical whispering gallery modes, which are confined close to the circumference of the disk. This allows us to further simplify the problem with a $r-\phi$ symmetry plane, which reduces the computational domain to a half (half disk in the case of a simple disk and a full-disk plus half silica layer for the double-disk structure). fig. S1 shows the computational domains used in both structures.

In order to calculate the optomechanical coupling rate the same mesh to resolve for both optical and mechanical wave equations is used. For the double disk structure the photoelastic contribution from silica to g_0 is not considered, since the slot-mode optical field is mainly confined in the air region between the silicon disks and close to the circumference. In fig. S1a-b we can see the kind of mesh used in the structures. In order to to improve convergence, we employ cubic interpolation functions for optical and mechanical modes. We also use rounded disk's corners (insets in fig. S1a-b), avoiding unrealistic optical fields that could impact the moving boundary overlap integrals.

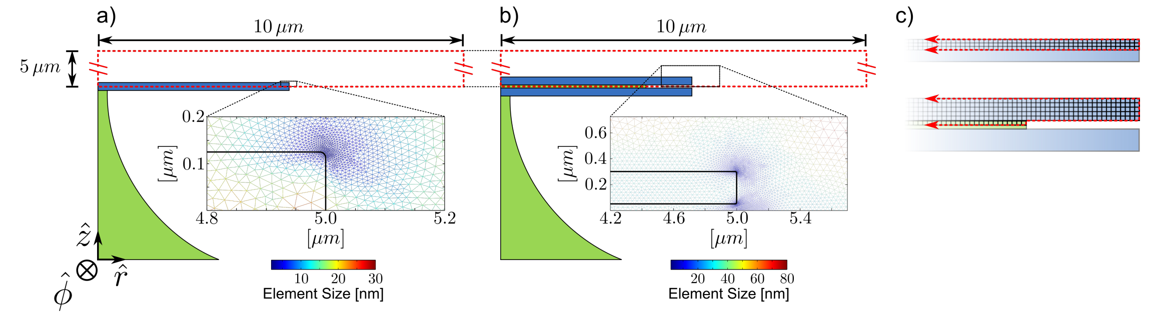

FIG. S1. Cross section of the computational domains (internal regions defined by the red dashed lines) in the single and double disks. **a)-b)** Modeling of the single and double disk to calculate the coupling between the optical and mechanical modes. White, blue and green regions represent the air, silicon and silica materials, respectively. In the inset figures show the kind of mesh and the element size. In the air domain, the element size enhances radialy with a maximum growth rate of 1.1. The top (and bottom in the double disk) right-corner is rounded, $r = 10\,nm$. **c)** Modeling of the single and double disk to calculate the mechanical dispersion ($\Omega/2\pi$ vs Azimuthal wavenumber - M). The cartesian black grid represent the kind of mesh that is used.

In order to calculate the modal mechanical dispersion of the structures the equation of motion (eq. (S14)) is solved by using rectangular finite elements (cartesian black grid inside of the red dashed line in fig. S1c). In both structures quadratic interpolation functions are used. Matlab Livelink was used to sweep azimuthal wavenumber - M parameter.

On the other hand, a perfect electric conductor boundary condition is assumed on the boundary of the computational domain to calculate the TM lowest-order mode. From mechanical point of view, both structures are simulated like a cantilever, i. e., the left-side boundary is fixed (part of the red dashed line along to the z-axis fig. S1a-b). In order to simulate dilatational and flexural modes in the single disk cavity the boundary conditions are $u_r \neq 0$, $u_z = 0$, $u_\phi \neq 0$, and $u_r = 0$, $u_z \neq 0$, $u_\phi = 0$ in the bottom boundary (red dashed lines along to the r-axis fig. S1c), respectively. In the double disk cavity only are applied the conditions: $u_r \neq 0$, $u_z = 0$, $u_\phi \neq 0$.

S3. CALCULATION OF THE OPTOMECHANICAL COUPLING RATE: g_0

In order to calculate g_0 the ansatz that is used to the eqs. (S4) and (S14) is given by,

$$\boldsymbol{E}_j(\boldsymbol{r}) = (E_j^{(r)}, E_j^{(\phi)} i, E_j^{(z)})\, e^{-im_j\phi}, \tag{S37}$$

$$\boldsymbol{u}(\boldsymbol{r}) = (u^{(r)}, u^{(\phi)} i, u^{(z)})\, e^{-iM\phi}, \tag{S38}$$

respectively, with $j =$ p, s. We also assume that the phase-matching condition is satisfied ($M = m_\mathrm{p} - m_\mathrm{s}$) and the backscattered Stokes mode as a complementary mode [7], i.e.,

$$E_\mathrm{s}^{(r)} = E_\mathrm{p}^{(r)} = E^{(r)}, \tag{S39}$$

$$E_\mathrm{s}^{(\phi)} = -E_\mathrm{p}^{(\phi)} = -E^{(\phi)}, \tag{S40}$$

$$E_\mathrm{s}^{(z)} = E_\mathrm{p}^{(z)} = E^{(z)}, \tag{S41}$$

The optomechanical coupling rate can be decomposed in two contributions, $g_{\rm mb}$ and $g_{\rm pe}$, below we detail our calculations for both contributions.

Moving-boundary contribution

For the moving boundary contribution, using eqs. (S37) to (S41) and eq. (S36) with the relation $g_{\rm mb} = g_{\rm om}^{\rm mb} x_{\rm zpf}$, we can break up the moving boundary contribution in three terms related to each optical field component,

$$g_{\rm mb} = \sum_{k=1}^{3} g_{\rm mb}^{(k)}, \tag{S42}$$

where,

$$g_{\rm mb}^{(k)} = -\frac{\omega_{\rm p}\, x_{\rm zpf}}{2} \oint_S u_\perp \rho_{\rm mb}^{(k)}\, dA, \tag{S43}$$

with the contributions to the optical weighting function $\rho_{\rm mb} = \sum_{k=1}^{3} \rho_{\rm mb}^{(k)}$ given by,

$$\rho_{\rm mb}^{(1)} = \delta\epsilon_{\rm mb} E_\parallel^2, \tag{S44}$$

$$\rho_{\rm mb}^{(2)} = -\delta\epsilon_{\rm mb} [E^{(\phi)}]^2, \tag{S45}$$

$$\rho_{\rm mb}^{(3)} = -\delta\epsilon_{\rm mb}^{-1} D_\perp^2, \tag{S46}$$

the normal and tangential field and displacement components are $u_\perp = u^{(r)} n_r + u^{(z)} n_z$, $E_\parallel = E^{(r)} t_r + E^{(z)} t_z$, $D_\perp = D^{(r)} n_r + D^{(z)} n_z$, $\hat{n} = (n_r, 0, n_z)$ and $\hat{t} = (t_r, 0, t_z)$. $\hat{n}$ and $\hat{t}$ are the normal and tangential unitary vectors in the transverse rz-plane, respectively. The minus signal in $\rho_{\rm mb}^{(2)}$ arise from of the Stokes ϕ-component in eq. (S40).

Figure S3 shows all the tangential and perpendicular electric field components and the contributions to the weighting function (eqs. (S44) to (S46)) for the lowest order TM mode in a single disk cavity. Interestingly, although $E_\perp^2$ is the largest optical field component, the dominant contribution to the optical weighting function is $\rho_{\rm mb}^{(2)}$, which is proportional to the azimuthal field component. The reason why the azimuthal field dominates over the vertical field is obvious if we rewrite,

$$\rho_{\rm mb}^{(3)} = \delta\epsilon_{\rm mb} E_\perp^2 \left[\frac{n_2}{n_1}\right]^2, \tag{S47}$$

where $n_1 = 3.5$ and $n_2 = 1$ are the refractive indexes of the single disk cavity and the region outside of cavity, respectively. Due to the factor $(n_2/n_1)^2 \approx 0.1$ in eq. (S47), the vertical component contribution is reduced by roughly one order of magnitude due to the high refractive index constrast.

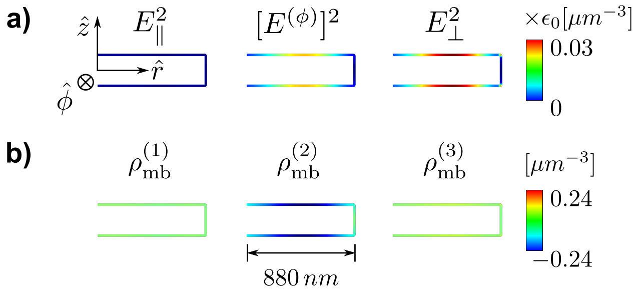

FIG. S2. Spatial distribution of the overlapping between the TM and surface (d_2, $\Omega/2\pi = 16.87\, GHz$) modes in the single disk. The mode, d_2, induces a **a)** strain ($\boldsymbol{S}$), **b)** permitivitty fluctuation ($\delta\boldsymbol{\epsilon}_{\rm pe}$) and a **c)** overlapping with the TM mode ($I_{\rm pe}^k$, $k = 1, .., 6$). As the strain is calculated from the unitary displacement $\boldsymbol{u}$, then units of the inverse to the length should be used.

In table S1a and table S1b we show each component of the moving-boundary contribution for two mechanical modes, the dilational mode d_2 (shown in main text's Figure 4h) and the whispering gallery mode w_{16} (shown in main text's Figure 4f).

$g_{\rm mb}^{(1)}$	$g_{\rm mb}^{(2)}$	$g_{\rm mb}^{(3)}$	$g_{\rm mb}$	$g_{\rm om}^{\rm mb}$
+2.5	−134	+15.3	−116.4	−323

(a) $\Omega/2\pi = 16.87$ GHz, $x_{\rm zpf} = 0.36$ fm, $m_{\rm eff} = 3.8$ pg

$g_{\rm mb}^{(1)}$	$g_{\rm mb}^{(2)}$	$g_{\rm mb}^{(3)}$	$g_{\rm mb}$	$g_{\rm om}^{\rm mb}$
−0.15	+6.45	−0.81	+5.5	+24

(b) $\Omega/2\pi = 24.34$ GHz, $x_{\rm zpf} = 0.23$ fm, $m_{\rm eff} = 6.3$ pg.

TABLE S1. Moving-boundary optomechanical coupling components ($\times 1/2\pi$) for the d_2 (a) and w_{16} (b) mechanical modes. Azimuthal number $M = 70$, $g_{\rm om}^{\rm mb}$ (in GHz/nm) and $g_{\rm mb}$ (in kHz).

Photo-elastic contribution

In order to grasp the nature of photoelastic component we substitute eqs. (S37) to (S41) in eq. (S35) and use the relation $g_{\rm pe} = g_{\rm om}^{\rm pe} x_{\rm zpf}$,

$$g_{\rm pe} = \sum_{k=1}^{6} g_{\rm pe}^{(k)}, \tag{S48}$$

where,

$$g_{\rm pe}^{(k)} = -\frac{\omega_{\rm p} x_{\rm zpf}}{2} \int_V I_{\rm pe}^{(k)} dV, \tag{S49}$$

and,

$$\begin{aligned} I_{\rm pe}^{(1)} &= \delta\epsilon_{\rm pe}^{rr} [E^{(r)}]^2, & I_{\rm pe}^{(4)} &= -2iE^{(\phi)} \delta\epsilon_{\rm pe}^{\phi z} E^{(z)}, \\ I_{\rm pe}^{(2)} &= -\delta\epsilon_{\rm pe}^{\phi\phi} [E^{(\phi)}]^2, & I_{\rm pe}^{(5)} &= 2E^{(r)} \delta\epsilon_{\rm pe}^{rz} E^{(z)}, \\ I_{\rm pe}^{(3)} &= \delta\epsilon_{\rm pe}^{zz} [E^{(z)}]^2, & I_{\rm pe}^{(6)} &= -2iE^{(r)} \delta\epsilon_{\rm pe}^{r\phi} E^{(\phi)}, \end{aligned} \tag{S50}$$

are the contributions to spatial overlap $I_{\rm pe} = \sum_{k=1}^{6} I_{\rm pe}^{(k)}$ and the dielectric perturbations due to photoelastic effect are given by,

$$\begin{aligned} \delta\epsilon_{\rm pe}^{rr} &= -\epsilon_0 n_1^4 (p_{11} S_{rr} + p_{12} [S_{\phi\phi} + S_{zz}]), & \delta\epsilon_{\rm pe}^{\phi z} &= -\epsilon_0 n_1^4 (p_{44} S_{\phi z}), \\ \delta\epsilon_{\rm pe}^{\phi\phi} &= -\epsilon_0 n_1^4 (p_{11} S_{\phi\phi} + p_{12} [S_{rr} + S_{zz}]), & \delta\epsilon_{\rm pe}^{rz} &= -\epsilon_0 n_1^4 (p_{44} S_{rz}), \\ \delta\epsilon_{\rm pe}^{zz} &= -\epsilon_0 n_1^4 (p_{11} S_{zz} + p_{12} [S_{rr} + S_{\phi\phi}]), & \delta\epsilon_{\rm pe}^{r\phi} &= -\epsilon_0 n_1^4 (p_{44} S_{r\phi}), \end{aligned} \tag{S51}$$

where each strain tensor component is calculated as,

$$\begin{aligned} S_{rr} &= \partial_r u^{(r)}, & S_{\phi z} &= \frac{i}{2} \left[\partial_z u^{(\phi)} - \frac{M u^{(z)}}{r} \right], \\ S_{\phi\phi} &= \frac{u^{(r)} + M u^{(\phi)}}{r}, & S_{rz} &= \frac{1}{2} \left[\partial_z u^{(r)} + \partial_r u^{(z)} \right], \\ S_{zz} &= \partial_z u^{(z)}, & S_{r\phi} &= \frac{i}{2} \left[-\frac{M u^{(r)}}{r} + \left[\partial_r - \frac{1}{r} \right] u^{(\phi)} \right]. \end{aligned} \tag{S52}$$

Similarly to $g_{\rm mb}$, $g_{\rm pe}$ is also real.

We also take the dilational mode d_2 (shown in the main text's figure 4h) and the whispering gallery mode w_{16} (shown in the main text's figure 4f) to understand the spatial overlap behavior of eq. (S50). In Figure S3 we show each contributions in eq. (S50), the strain components (eq. (S52)), and the dielectric perturbations (eq. (S51)). The spatial overlap $I_{\rm pe}^{(3)}$ is dominant for the d_2 modes (fig. S3), whereas the $I_{\rm pe}^{(2)}$ is dominant for the w_{16} mode (fig. S4c). A similar correspondence can be observed both in the strain and dielectric perturbations.

The peaked g_0 contribution of the whispering mode group in the main text figure 4a is caused by the presence of a net positive azimuthal strain region close to the circumference of the single disk cavity. In fig. S4d we show this behavior in detail for the w_{16} mode. The physical origin of this positive net strain region can be traced by exploring

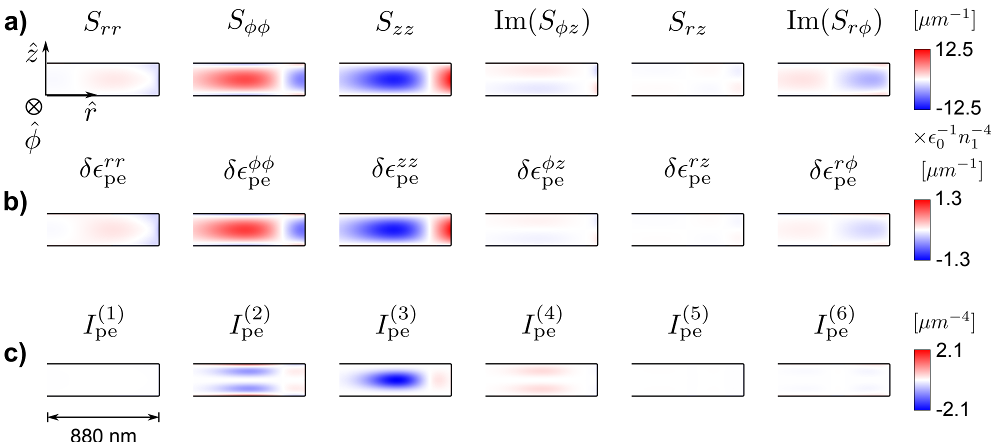

FIG. S3. Spatial distribution of the overlapping between the TM and surface (d_2, $\Omega/2\pi = 16.87\,GHz$) modes in the single disk. The mode, d_2, induces a **a)** strain ($\boldsymbol{S}$), **b)** permitivitty fluctuation ($\delta\boldsymbol{\epsilon}_{\mathrm{pe}}$) and a **c)** overlap integrand (I_{pe}^{k}). As the strain is calculated from the unitary displacement it has units L^{-1}.

the analytical expression for $S_{\phi\phi}$ obtained for an infinite elastic cylinder.

$$S_{\phi\phi} = \underbrace{\frac{u_{\mathrm{l}}^{(r)} + M u_{\mathrm{l}}^{(\phi)}}{r}}_{S_{\phi\phi}^{\mathrm{l}}} + \underbrace{\frac{u_{\mathrm{t}}^{(r)} + M u_{\mathrm{t}}^{(\phi)}}{r}}_{S_{\phi\phi}^{\mathrm{t}}}, \tag{S53}$$

where,

$$u_{\mathrm{l}}^{(r)} = -\frac{\tilde{\Omega}}{\eta} J_M' \left(\frac{\tilde{r}\tilde{\Omega}}{\eta}\right), \qquad u_{\mathrm{t}}^{(r)} = \frac{M f(\tilde{\Omega}) J_M(\tilde{r}\tilde{\Omega})}{\tilde{r}}, \tag{S54}$$

$$u_{\mathrm{l}}^{(\phi)} = \frac{M}{\tilde{r}} J_M \left(\frac{\tilde{r}\tilde{\Omega}}{\eta}\right), \qquad u_{\mathrm{t}}^{(\phi)} = -\tilde{\Omega} f(\tilde{\Omega}) J_M'(\tilde{r}\tilde{\Omega}), \tag{S55}$$

are the contributions from the longitudinal (l) and transverse (t) waves to each displacement component [8],

$$f(\tilde{\Omega}) = \frac{1}{\eta^2} \frac{J_{M-2}\left(\frac{\tilde{\Omega}}{\eta}\right) - J_{M+2}\left(\frac{\tilde{\Omega}}{\eta}\right)}{J_{M-2}(\tilde{\Omega}) + J_{M+2}(\tilde{\Omega})}, \tag{S56}$$

where J_M is the Bessel function of the first kind of order M, $\tilde{\Omega} = \frac{\Omega_0^{\mathrm{c}} a}{V_{\mathrm{t}}}$ is the normalized angular frequency; Ω_0^{c} is the angular frequency, a is the cylinder radius and the transverse bulk velocity is V_{t}, $\eta = \frac{V_{\mathrm{l}}}{V_{\mathrm{t}}}$; V_{l} is the longitudinal bulk velocity and $\tilde{r} = r/a$ is the normalized radius.

There is a surprisingly good agreement between the analytic (blue solid line) mode profile and the actual numerical mode for the microdisk (blue hollow circles) in the fig. S4d. The analytical solution has an explicit contribution from the longitudinal and tranverse propagation velocities. The slowly varying contribution, is due to the smaller radial wavevector associated with the longitudinal wave. Indeed with we plot just this contribution in the analytical solution we can precisely reproduce the bump observed in the numerical solution(fig. S4e). Therefore we attribute the slowly varying positive net strain to the contrasting velocities of transverse and longitudinal acoustic waves in Si. This is fundamental to explain the behavior observed for w-mode group in main text's Figure 4(a,b).

In table S2b and table S2a we also show each component of the photo-elastic contribution for the two mechanical modes discussed in fig. S3 and fig. S4, the dilational mode d_2 and the whispering gallery mode w_{16}. In both tables the dominant contributions (values in blue color) reflect the overlaps functions, as expected. We see that $g_{\mathrm{pe}}(d_2)$ is 67% greater than $g_{\mathrm{pe}}(w_{16})$, which it is not true for the dominant contributions $g_{\mathrm{pe}}^{(3)}(d_2)$ and $g_{\mathrm{pe}}^{(2)}(w_{16})$.

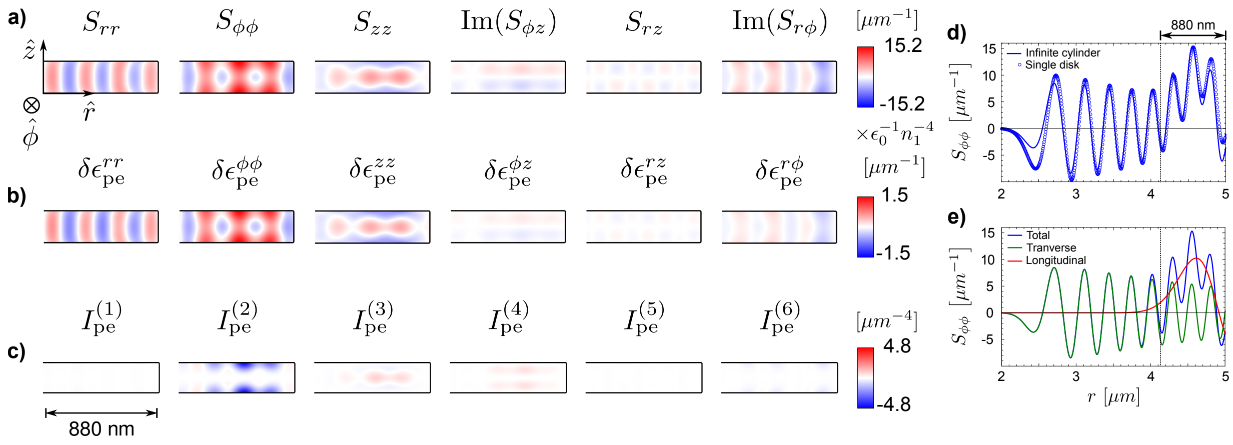

FIG. S4. Spatial distribution of the overlap integrals between the TM and w_{16} ($\Omega/2\pi = 24.34\,GHz$) modes in the single disk. **a)** Mechanical strain ($\boldsymbol{S}$), **b)** Permitivitty perturbation ($\delta\boldsymbol{\epsilon}_{\rm pe}$) and a **c)** overlap integrands ($I_{\rm pe}^{k}$). **d)** Radial behavior of $S_{\phi\phi}$. Analytic (blue solid line) and simulation (blue hollow circles) data are shown to the infinite cylinder and the single disk, respectively. The single disk linecut is taken along the top plane. **e)** Analytic curves of the radial behavior of the transverse (green solid line) and longitudinal (red solid line) contributions to $S_{\phi\phi}$ (blue solid line) to the infinite cylinder.

$g_{\rm pe}^{(1)}$	$g_{\rm pe}^{(2)}$	$g_{\rm pe}^{(3)}$	$g_{\rm pe}^{(4)}$	$g_{\rm pe}^{(5)}$	$g_{\rm pe}^{(6)}$	$g_{\rm pe}$	$g_{\rm om}^{\rm pe}$
+0.02	+22.2	+78.8	−14.7	−0.3	+1.0	+87.2	+242

(a) $\Omega/2\pi = 16.87$ GHz, $x_{\rm zpf} = 0.36$ fm, $m_{eff} = 3.8$ pg

$g_{\rm pe}^{(1)}$	$g_{\rm pe}^{(2)}$	$g_{\rm pe}^{(3)}$	$g_{\rm pe}^{(4)}$	$g_{\rm pe}^{(5)}$	$g_{\rm pe}^{(6)}$	$g_{\rm pe}$	$g_{\rm om}^{\rm pe}$
+0.2	+79.3	−7.9	−16.6	−0.4	+6.6	+61.2	+266

(b) $\Omega/2\pi = 24.34$ GHz, $x_{\rm zpf} = 0.23$ fm, $m_{eff} = 6.3$ pg

TABLE S2. Photo-elastic optomechanical coupling components ($\times 1/2\pi$) for the d_2 (a) and w_{16} (b) mechanical modes. Azimuthal number $M = 70$, $g_{\rm om}^{\rm pe}$ (in GHz/nm) and $g_{\rm pe}$ (in kHz).

S4. BRILLOUIN LASING THRESHOLD

In order to calculate the power threshold we take the eqs. (S31) to (S33) and add the losses ($\kappa_{\rm e}$, $\kappa_{\rm p}$, $\kappa_{\rm s}$, Γ), the normalized power amplitude ($s_{\rm p} \to \frac{s_{\rm p}}{\sqrt{\hbar\omega_{\rm p}}}$) and considering that $g_0 \to g_0^{\rm c} = (g_0^{\rm c})^*$, where $g_0^{\rm c}$ is the vacuum optomechanical coupling rate for the compound cavity,

$$\dot{a}_{\rm p} = \chi_{\rm p}^{-1} a_{\rm p} - i\, g_0^{\rm c}\, b\, a_{\rm s} + \sqrt{\kappa_{\rm e}} s_{\rm p}, \tag{S57}$$

$$\dot{a}_{\rm s} = \chi_{\rm s}^{-1} a_{\rm s} - i\, g_0^{\rm c}\, b^* a_{\rm p}, \tag{S58}$$

$$\dot{b} = \chi_{\rm m}^{-1} b - i\, g_0^{\rm c}\, a_{\rm p} a_{\rm s}^*, \tag{S59}$$

in which $\chi_{\rm p}^{-1} = i\Delta_{\rm p} + \frac{\kappa_{\rm p}}{2}$, $\chi_{\rm s}^{-1} = i\Delta_{\rm s} + \frac{\kappa_{\rm s}}{2}$ and $\chi_{\rm m}^{-1} = i\Delta_{\rm m} + \frac{\Gamma}{2}$. Now following [9], the steady-state in the eqs. (S57) and (S59) leads to,

$$\dot{a}_{\rm s} = \left[\chi_{\rm s}^{-1} - \frac{\kappa_{\rm e}|s_{\rm p}|^2 (g_0^{\rm c})^2}{[\chi_{\rm m}^{-1}]^* |\chi_{\rm p}^{-1}|^2} \left|1 + \frac{|a_{\rm s}|^2 (g_0^{\rm c})^2}{\chi_{\rm m}^{-1}\chi_{\rm p}^{-1}}\right|^{-2}\right] a_{\rm s}. \tag{S60}$$

The threshod condition occurs when the mechanically-induced gain is high enough to ensure a steady-state for eq. (S60), this is achieved by requiring the term between parentheses to be zero,

$$|s_{\rm p}|^2 > \frac{[\chi_{\rm m}^{-1}]^* \chi_{\rm s}^{-1} |\chi_{\rm p}^{-1}|^2}{\kappa_{\rm e} (g_0^{\rm c})^2}. \tag{S61}$$

From the eq. (S61) we have a product between two complex variables: $[\chi_{\rm m}^{-1}]^* \chi_{\rm s}^{-1}$. In order to understand the nature of this product we come back to the expression between parentheses in the eq. (S60) in the steady-state and rewrite,

$$\chi_{\rm s}^{-1} = \frac{\kappa_{\rm e}|s_{\rm p}|^2 (g_0^{\rm c})^2}{|\chi_{\rm m}^{-1}|^2 |\chi_{\rm p}^{-1}|^2} \left|1 + \frac{|a_{\rm s}|^2 (g_0^{\rm c})^2}{\chi_{\rm m}^{-1}\chi_{\rm p}^{-1}}\right|^{-2} \chi_{\rm m}^{-1}. \tag{S62}$$

By using the expressions to $\chi_{\rm s}^{-1}$ and $\chi_{\rm m}^{-1}$ results,

$$\frac{\Delta_{\rm s}}{\kappa_{\rm s}} = \frac{\Delta_{\rm m}}{\Gamma}. \tag{S63}$$

Using the eq. (S63) and the expression to $\chi_{\rm p}^{-1}$ in the threshold condition (eq. (S61)) we obtain $|s_{\rm p}|^2 > P_{\rm th}$,

$$P_{\rm th} = \frac{\hbar\omega_{\rm p}\kappa_{\rm p}^2}{4\,\mathcal{C}\kappa_{\rm e}}\left[1+\left(\frac{\Delta_{\rm s}}{\kappa_{\rm s}/2}\right)^2\right]\left[1+\left(\frac{\Delta_{\rm p}}{\kappa_{\rm p}/2}\right)^2\right], \tag{S64}$$

in which $\mathcal{C} = \frac{4(g_0^{\rm c})^2}{\Gamma\kappa_{\rm s}}$ is the so-called single-photon cooperativity.

---

[1] Agarwal, G. S. & Jha, S. S. Multimode phonon cooling via three-wave parametric interactions with optical fields. *Phys. Rev. A* **88**, 013815 (2013).

[2] Boyd, R. W. *Nonlinear Optics* (Elsevier Science, Burlington, MA, 2013).

[3] Panofsky, W. & Phillips, M. *Classical Electricity and Magnetism: Second Edition*. Dover Books on Physics (Dover Publications, 2012).

[4] Rakich, P. T., Reinke, C., Camacho, R., Davids, P. & Wang, Z. Giant enhancement of stimulated Brillouin scattering in the subwavelength limit. *Phys. Rev. X* **2**, 011008 (2012).

[5] Wolff, C., Steel, M. J., Eggleton, B. J. & Poulton, C. G. Stimulated Brillouin scattering in integrated photonic waveguides: forces, scattering mechanisms, and coupled-mode analysis. *Phys. Rev. A* **92**, 013836 (2015).

[6] Van Laer, R., Baets, R. & Van Thourhout, D. Unifying brillouin scattering and cavity optomechanics. *Phys. Rev. A* **93**, 053828 (2016).

[7] Mrozowski, M. *Guided Electromagnetic Waves: Properties and Analysis*. Computer methods in electromagnetics series (Research Studies Press, 1997).

[8] Dmitriev, A., Gritsenko, D. & Mitrofanov, V. Surface vibrational modes in disk-shaped resonators. *Ultrasonics* **54**, 905 – 913 (2014).

[9] Matsko, A. B., Ilchenko, V. S., Savchenkov, A. A. & Maleki, L. Highly nondegenerate all-resonant optical parametric oscillator. *Phys. Rev. A* **66**, 043814 (2002).
